# Identification of KIF23 as a Prognostic Biomarker Associated With Progression of Clear Cell Renal Cell Carcinoma

**DOI:** 10.3389/fcell.2022.839821

**Published:** 2022-04-11

**Authors:** Zonglong Wu, Yimeng Song, Yaqian Wu, Liyuan Ge, Zhuo Liu, Tan Du, Shudong Zhang, Lulin Ma

**Affiliations:** Department of Urology, Peking University Third Hospital, Beijing, China

**Keywords:** renal cell carcinoma, kinesin family member 23, metastasis-associated genes, prognosis signature, Wnt/β-catenin signaling pathway

## Abstract

About 3% of adult cancers are caused by renal cell carcinoma (RCC) and its pathogenesis remains elusive. Among RCC, clear cell renal cell carcinoma (ccRCC) is the predominant histological subtype. Resistance to conventional treatments leaves few treatment options for advanced ccRCC. Although the transcriptome profile of primary ccRCC has been comprehensively summarized, the transcriptome profile of metastatic ccRCC is still lacking. In this study we identified a list of metastasis-related genes and constructing a metastasis-associated prognostic gene signature. By analyzing data from GSE85258 and GSE105288 datasets, 74 genes were identified as metastasis-related genes. To construct prognostic features, we downloaded the expression data of ccRCC from the Cancer Genome Atlas (TCGA). Metastasis-associated genes were initially selected through the LASSO Cox regression analysis and 12 metastasis-related were included to construct prognostic model. Transcriptome profile, patient prognosis, and immune cell infiltration characteristics differed between low- and high-risk groups after grouping according to median risk score. Through explored the functions of differentially expressed genes (DEGs) between the two groups. Kinesin family member 23 (KIF23) was identified as a prognostic marker in ccRCC patients. Furthermore, inhibition of KIF23 expression reduced the proliferation, migration and invasion of ccRCC cells. We further demonstrated that KIF23 promote nuclear translocation of β-catenin in ccRCC cells, which provides novel insight into the functions and molecular machinery of KIF23 in ccRCC.

## Introduction

About 3% of adult cancers are caused by renal cell carcinoma (RCC) and its pathogenesis remains elusive. ccRCC is the predominant histological subtype and is prone to metastases. Patients with metastatic ccRCC have a poor prognosis, with a 5-year overall survival (OS) rate is 8–12% (Choueiri and Motzer, 2017). ccRCC is a highly vascularized malignant tumor. Therapies targeting angiogenesis are effective in tumor regression. However, most patients treated with targeted agents often develop resistance within 1 year of treatment, which warrants new treatment strategies (Rini and Atkins, 2009). ccRCC is a highly heterogeneous disease with complex molecular signatures and biological characteristics (Iacovelli et al., 2014). Several biological factors may affect the biological process and treatment response. An accurate understanding of the characteristics of ccRCC will help in the management of ccRCC patients. Therefore, stratifying the risk of patients to discover their molecular characteristics and predict response to personalized drug treatment is of great significance.

Due to insidious progression, nearly one-third of ccRCC patients have metastases at initial medical attention, approximately 60% of non-metastatic ccRCC patients develop lung, bone or other organ metastases within 2–3 years (Park and Eisen, 2007; Casuscelli et al., 2019). Metastasis is the main reason of ccRCC-related deaths. Although some patients benefit from immunotherapy, ccRCC remains an incurable disease for the majority of patients (Escudier et al., 2020). Therefore, exploring a new biomarker of tumor metastasis is crucial for monitoring metastasis and treatment. Although somatic mutation and transcriptome profiles of primary ccRCC have been comprehensively summarized in the Cancer Genome Atlas (TCGA) database, the transcriptome and genome profiles of metastatic ccRCC are largely lacking. Analyzing the differential gene expression between metastatic and primary tumors helps to determine factors influencing metastasis and predict prognosis in advance.

In this study, we compared transcriptome profiles between metastatic and primary tumors to identify genes associated with ccRCC metastases. Furthermore, prognostic signatures of metastasis-related genes were constructed. After stratifying ccRCC patients according to risk score, transcriptional profiles, patient prognosis, and immune cell infiltration characteristics differed between low- and high-risk groups. Finally, we identified kinesin family member 23 (KIF23) as an important gene that drives ccRCC progression and affects patient prognosis. Knockdown of KIF23 expression in ccRCC cells attenuated cell proliferation migration and invasion, and reduced nuclear translocation of β-catenin.

## Materials and Methods

### Identification of Metastasis-Associated Genes in Clear Cell Renal Cell Carcinoma

Microarray datasets GSE85258 and GSE105288 in the Gene Expression Omnibus (GEO) database were used to analyze the transcriptomic signature of metastatic ccRCC. The GSE85258 dataset contained 15 primary tumors and 16 metastatic tumor samples. The GSE105288 dataset contained nine primary tumors and 26 metastatic tumor samples. By GEO2R tool analysis, genes with fold change (FC) > 1.5 and *p* < 0.05 in metastatic ccRCC compared with primary tumors were defined as metastasis-related genes. Next, we obtained gene expression profiles of 530 ccRCCs from the TCGA data portal and analyzed them by the limma package. The screening criteria for differentially expressed genes (DEGs) were *p* < 0.05 and FC > 2. The Ggplot2 and pheatmap packages were used for volcano plots and heatmaps generation.

### Construction of Clinical Prognostic Model

The R package “glmnet” performed LASSO regression analysis to identify key prognostic genes. One standard error of the optimal penalty parameter λ value after cross-validation was used to build the simplest gene feature model. The risk score was determined by calculating the sum of the product of each gene expression level and its corresponding coefficient according to the risk score formula. Next, patients were stratified into two groups based on the median risk score. Differences in survival time between the two groups were estimated by the Kaplan-Meier method. Time-related receiver operating characteristic (ROC) analysis was used to verify the prediction accuracy of the model.

### CIBERSORT Analysis

CIBERSORT analysis tool was used to infers the proportion of infiltrating immune cells based on cell-type-specific gene expression profiles from the mixed cell data. Inferred results were considered accurate at *p* < 0.05.

### Functional Enrichment Analysis

ClusterProfiler package performed Gene Ontology (GO) and the Kyoto encyclopedia of genes and genomes (KEGG) pathway analysis (Yu et al., 2012). The PPI network was retrieved by the STRING database and reconstructed by Cytoscape software (Shannon et al., 2003; Szklarczyk et al., 2015). Cytohubba was used to analysis and select top 10 hub genes.

### Gene Set Enrichment Analysis Analysis

The C2 kegg gene set was used for GSEA enrichment analysis and FDR values <0.01 were considered significantly enriched.

### Clear Cell Renal Cell Carcinoma Specimens and Cell Culture

The ccRCC tissues and normal samples of 10 ccRCC patients involved in this study were obtained from Peking University Third Hospital after approval by the Ethics Committee of Peking University Third Hospital. The ccRCC cell lines A498, caki-1, caki-2, 786-O and OS-RC2 and the immortalized human kidney cell line HK-2 were purchased from the American Type Culture Collection. 1640 medium (Biological Industries) containing 10% fetal bovine serum (FBS) (Biological Industries) was used to culture cells.

### Synthesis of Small Interfering RNA and Transfection of Cells

Cells were transfected with specific siRNAs targeting KIF23 (si-KIF23). si-KIF23 were purchased from GenePharma Biotech (Shanghai, China). The siRNA sequence is:

si-KIF23-1 sense: 5′-GGU​CCC​AAA​CGA​ACC​UUA​ATT-3′, antisense: 5′-UUA​AGG​UUC​GUU​UGG​GAC​CTT-3′;
si-KIF23-2 sense: 5′- GCU​AUU​GUU​ACC​GAA​CCU​ATT-3′, antisense: 5′- UAG​GUU​CGG​UAA​CAA​UAG​CTT-3′;
si-KIF23-3 sense: 5′-CCU​CAU​GCC​AUC​ACA​GUA​UTT-3′, antisense: 5′- AUA​CUG​UGA​UGG​CAU​GAG​GTT-3′;
si-N.C sense: 5′-UUC​UCC​GAA​CGU​GUC​ACG​UTT-3′, antisense: 5′-ACG​UGA​CAC​GUU​CGG​AGA​ATT-3′.

Lipofectamine 2000 transfection reagent (Invitrogen, Carlsbad, CA, United States) was used to transfect cells following the manufacturer’s instructions.

### Cell Proliferation Assay

2 × 10^3^ ccRCC cells were cultured in a 96-well plate, and cell proliferation changes were assessed by a Cell Counting Kit 8 (CCK-8) (Beyotime, Shanghai, China) at 24, 48, and 72 h after si-KIF23 transfection. Ethynyl deoxyuridine (EdU) kit (Ribobio, Guangzhou, China) was used to perform EdU assay following the manufacturer’s instructions.

### Transwell Migration and Invasion Assays

100 μl of serum-free medium with 2 × 10^4^ ccRCC cells was added into the upper chamber of a transwell migration chamber (8-μm pore size, Costar, New York). The lower chamber was filled with medium containing 20% FBS. After 24 h, cells were fixed with 4% PFA and stained with crystal violet. Cells were counted under a microscope in five random fields after removal of cells on the upper surface. For invasion assay, upper chamber was coated with Matrigel (354480, Corning) before seeded cells.

### Wound-Healing Assay

The cells with different treatments were seeded in six-well plates, starved for 24 h, and wounded with a pipette tip. Then replace with fresh medium. After 24 h, the wound closure process was observed and photographed.

### Western Blot and Antibodies

RIPA buffer (Beyotime) was used for cell and tissue lysis. 10% sodium dodecyl sulfate-polyacrylamide gel electrophoresis (SDS-PAGE) was used for protein separation. Then, proteins were transferred onto polyvinylidene difluoride (PVDF) membrane (Millipore, Billerica, MA, United States) by electroblotting for 90 min. After blocked in 5% non-fat dry milk for 2 h, the PVDF membranes incubated with primary antibodies at 4°C overnight, followed by the secondary antibody for at least 1 h. The blots were then visualized using a Western Blotting Luminol Reagent. The primary antibodies of KIF23 (1:1000, Proteintech, 28587-1-AP), β-catenin (1:1000, Cell Signaling Technology, 8480), GSK-3β (1:1000, Cell Signaling Technology, 12456), p-GSK-3β (1:1000, Cell Signaling Technology, 5558), c-Myc (1:1000, Cell Signaling Technology, 18583) and GAPDH (1:2000, Proteintech, 10494-1-AP). Cytoplasmic protein extraction kit (Beyotime) was used to extract nuclear proteins following the manufacturer’s manual.

### Immunohistochemistry and Immunofluorescence Analysis

ccRCC tissue sections were deparaffinized and the antigen was retrieved, followed by blocking with 5% bovine serum albumin. Next, ccRCC tissue sections were incubated with KIF23 primary antibody (1:100, Proteintech, 28587-1-AP) at 4°C overnight, a peroxidase-conjugated secondary antibody was used to detect antigen-antibody complexes. Thereafter, a color reaction was conducted using a 3,3′-Diaminobenzidine (DAB) substrate kit (ZsBio). For immunofluorescence staining, ccRCC cells were seeded on coverslips and fixed in 4% PFA. Then, cells were washed and treated with 0.25% Triton X-100 for 15 min. After blocked by 5% donkey serum at room temperature for 30 min, cells were incubated with β-catenin (1:100, Cell Signaling Technology, 8480) at 4°C overnight. Alexa Fluor 594 conjugated secondary antibody (1:500; Yeasen) was used to detect antigen-antibody complexes. 4′,6-Diamidino-2-phenylindole (DAPI) was used for the staining of nucleus.

### Statistical Analysis

R (version 3.6) and GraphPad Prism software were used to execute statistical analysis. Differences between groups were statistically compared using the student’s t test or two-way ANOVA. Each experiment was performed in triplicate, and values were expressed as mean ± standard deviation. A *p*-value <0.05 was regarded statistically significant. Survival curves were obtained by the Kaplan-Meier method and compared by log-rank tests. And levels of significance were presented at *p < 0.05, **p < 0.01, ***p < 0.001 and ****p < 0.0001 respectively.

## Result

### Identification of Metastasis-Associated Genes

**FIGURE 1:**
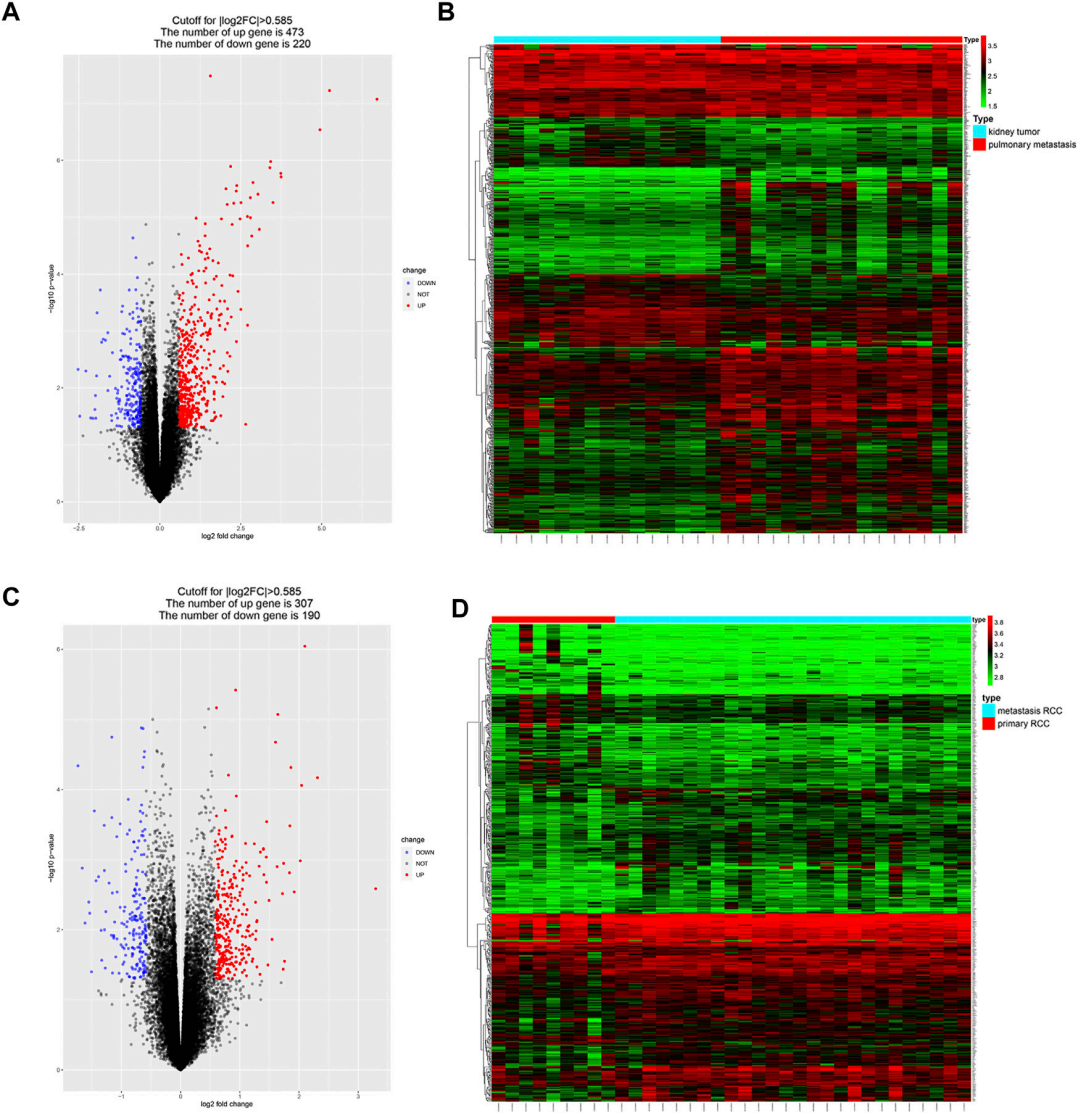
Identification of metastasis-associated genes in ccRCC. **(A–D)** Volcano plots and heatmaps of the DEGs in GSE85258 and GSE105288.

**FIGURE 2:**
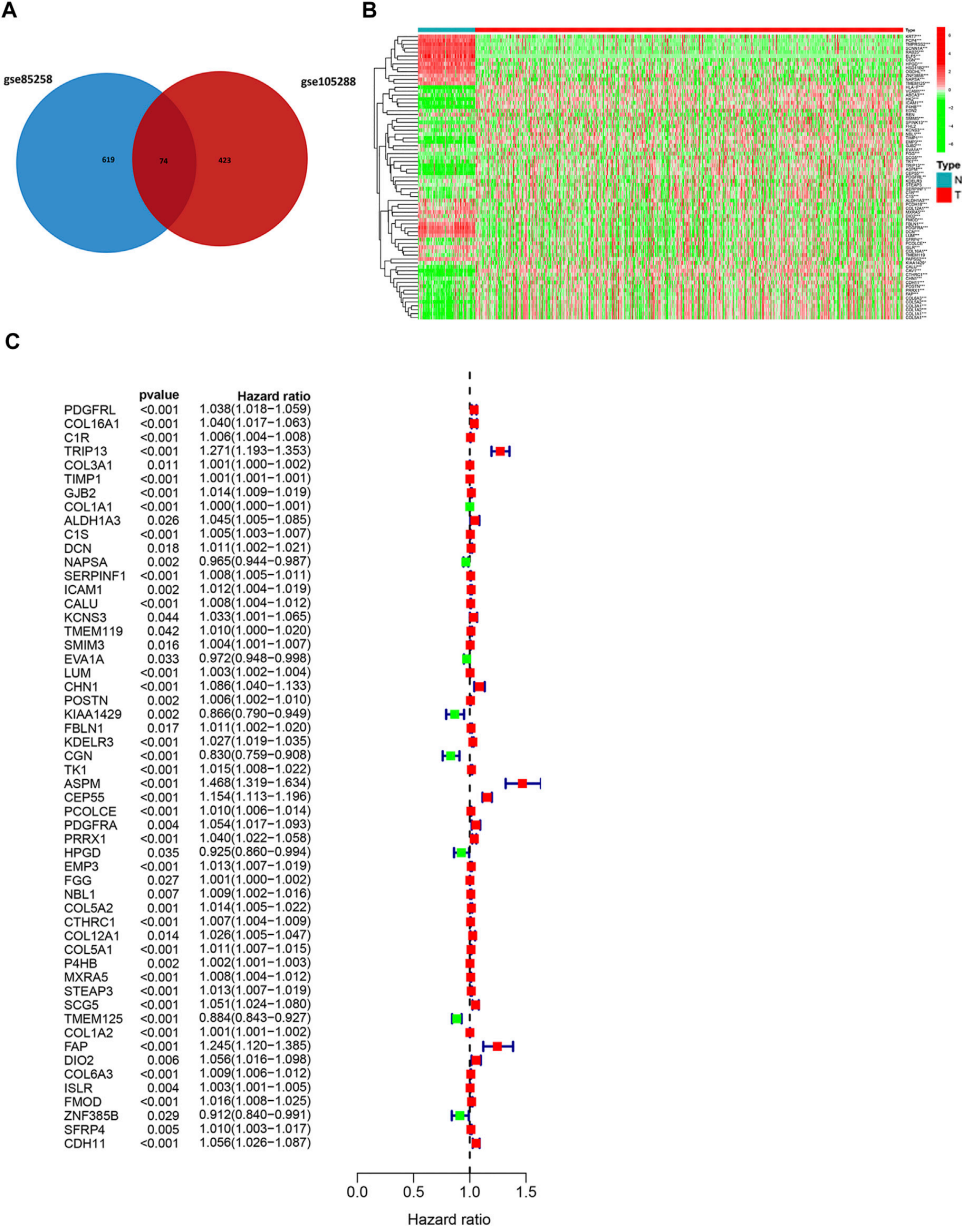
Expression profile of metastasis-associated genes in TCGA dataset. **(A)** Venn diagram of metastasis-associated DEGs. **(B)** Heatmap of metastasis-associated genes in TCGA ccRCC dataset. **(C)** Univariate Cox regression analysis.

DEGs between metastatic and primary tumors were analyzed by the GEO2R tool with FC > 1.5 and *p* < 0.05 as screening criteria. 693 genes (473 up and 220 down) were identified as DEGs in GSE85258.497 genes (307 up and 190 down) were identified as DEGs in GSE105288 (Figures 1A–D). Venn diagrams were used to integrate GSE85258 and GSE105288 datasets, and 74 DEGs were identified as metastasis-related genes (Figure 2A). To validate these metastasis-related genes, the ccRCC dataset from TCGA was downloaded and analyzed. The expression levels of these DEGs were visualized using a heatmap (Figure 2B). Univariate Cox proportional hazards regression analysis revealed 54 genes significantly associated with OS in ccRCC patients (Figure 2C). Forty seven genes with a hazard ratio (HR) greater than one were identified as “high risk factors”, and seven genes with HR < 1 were considered protective factors.

### Construction of a Prognostic Signature

**FIGURE 3:**
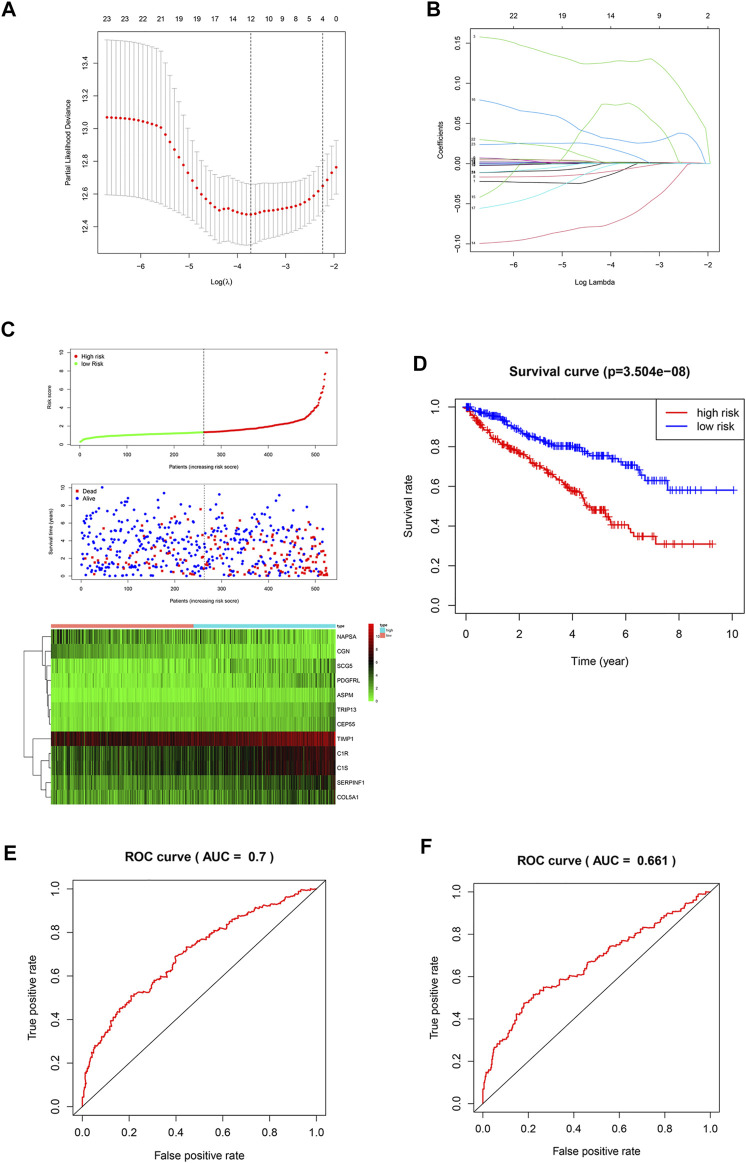
Construction of a prognostic signature. **(A,B)** LASSO analysis with a minimum lambda value. **(C)** Risk score, patient survival status distribution and expression heatmaps for 12 metastasis-associated prognostic risk signatures. **(D)** Kaplan-Meier curves of the low- and high-risk groups (*p* < 0.001). **(E,F)** ROC curve for 3- and 5-year survival predictions in patients with ccRCC based on the risk score.

It is difficult for a single metastasis-associated gene to accurately predict patient prognosis. Thus, development of a multi-gene combined prediction model is essential. To construct the prognostic model, the metastasis-associated genes were used to LASSO Cox regression analysis, and the regression coefficients were calculated as shown in Figure 3A. The best performing prognostic model was obtained when 12 genes were included (Figure 3B). The risk score for each patient is calculated from the expression levels of 12 genes: risk score = (−0.0072 * PDGFRL) + (0.0012 * C1R) + (0.1278 * TRIP13) + (0.0004 * TIMP1) + (0.0016 * C1S) + (−0.0072 * NAPSA) + (0.0011 * SERPINF1) + (−0.06808 * CGN) + (0.07435 * ASPM) + (0.02810 * CEP55) + (0.00039 * COL5A1) + (0.01375 * SCG5). Then, patients were divided into low-risk (*n* = 263) and high-risk (*n* = 262) groups based on their risk scores (Figure 3C). Patients in high-risk group have a worse prognosis (Figure 3D). The area under curve (AUC) value for metastasis-associated prognostic gene signature was 0.7 and 0.661 for 3- and 5-year survival rates, respectively (Figures 3E,F).

### Identification of Immune Cell Infiltration Characteristics of Each Group

**FIGURE 4:**
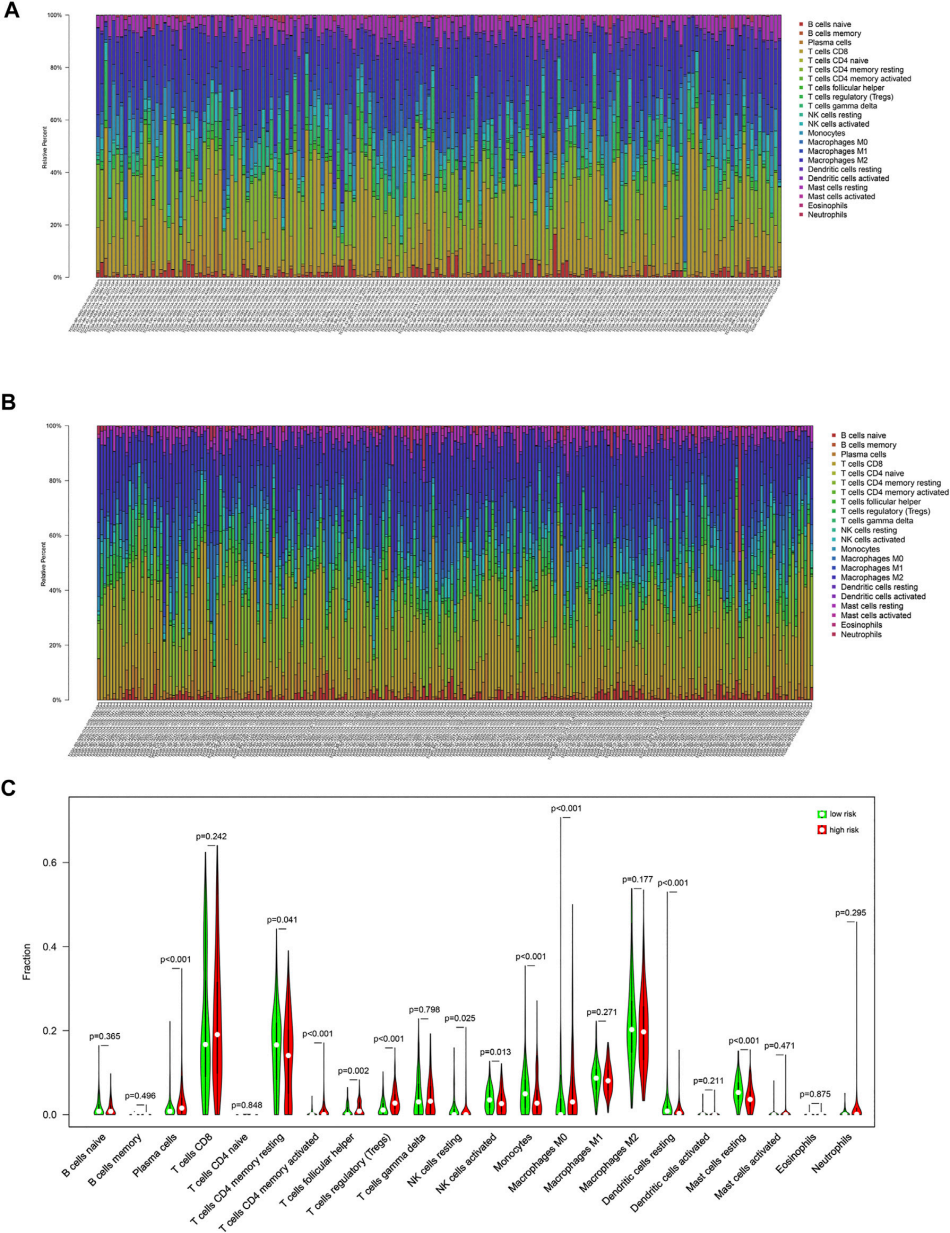
Immune cell infiltration signatures of each group. **(A)** The immune cell infiltration signatures in the low-risk group. **(B)** The immune cell infiltration signatures in the high-risk group. **(C)** Differences in immune cell infiltration characteristics between low-risk and high-risk populations.

In this study, CIBERSORT algorithm was used to estimate the proportion of infiltrating immune cells in tumor tissue. Immune cell infiltration signatures of the low-risk and the high-risk groups are showed in Figures 4A,B. There were higher proportions of resting memory CD4 T cells, activated natural killer (NK) cells, monocytes, resting dendritic cells (DCs) and resting mast cells in the low-risk group. Plasma cells, regulatory T cells, and M0 macrophages were higher in the high-risk group (Figure 4C).

### Differences in Gene Expression Between the Low- and High-Risk Groups

**FIGURE 5:**
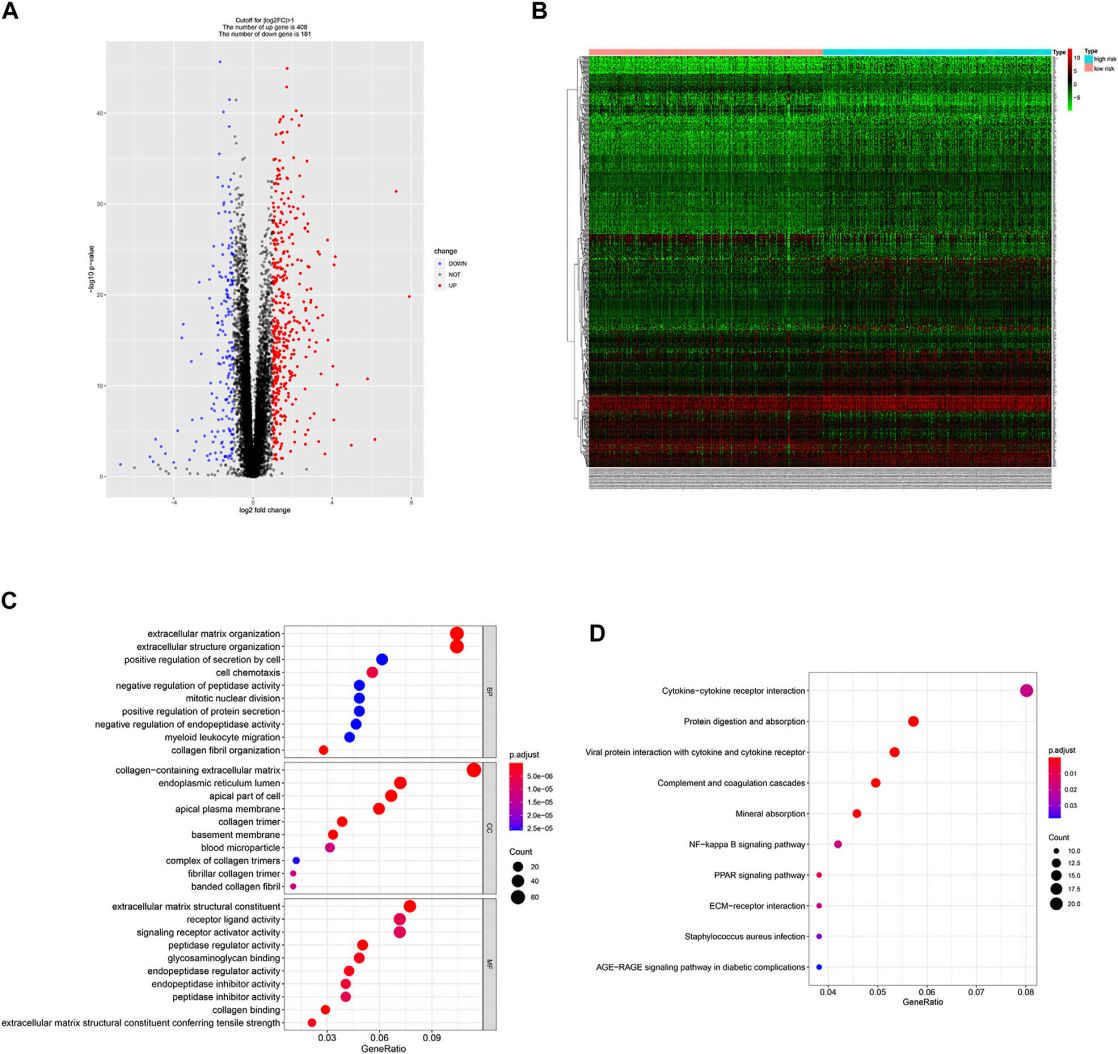
DEGs identification and functional enrichment analysis. **(A,B)** Volcano plot and heatmap of 588 DEGs between low- and high-risk group. **(C,D)** Function enrichment analysis of DEGs.

Since the two groups showed variations in gene expression profiles and prognosis, gene expression patterns between the groups were analyzed and 588 DEGs were identified (408 and 181 genes were up-regulated in the high- and low-risk groups, respectively, Figures 5A,B).

### Function Enrichment Analysis

**FIGURE 6:**
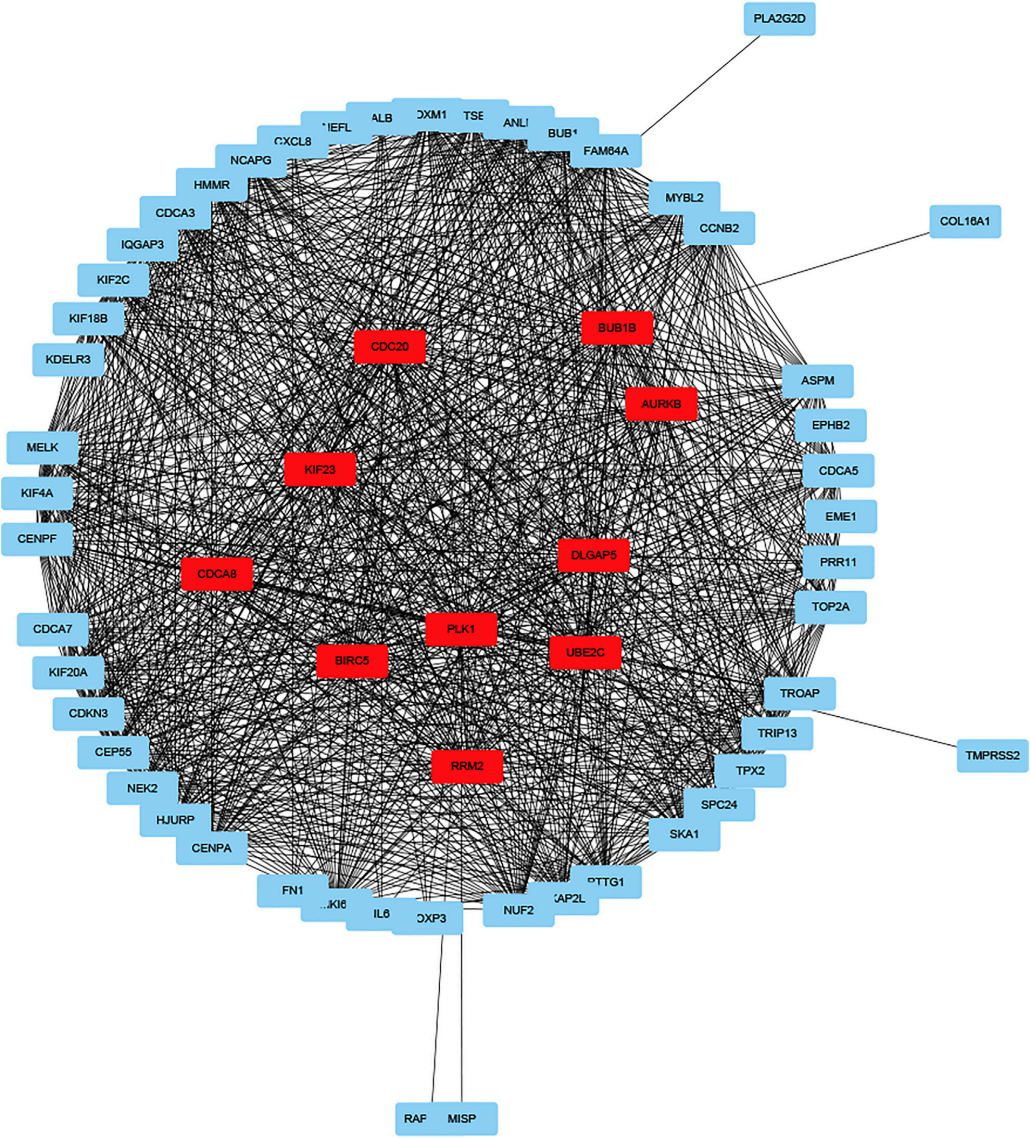
Protein-protein interaction networks of DEGs.

GO enrichment analysis showed that DEGs were involved in “extracellular matrix organization”, “extracellular structure organization” and “positive regulation of secretion by cell” in the “biological process” category. “Collagen-containing extracellular matrix” was enriched in the “cellular components” category and “extracellular matrix structural constituent” was significantly enriched in the “molecular function” category. KEGG pathway enrichment analysis results indicated that “cytokine-cytokine receptor interaction” and “NF-kappa B signaling pathway” are significantly enriched (Figures 5C,D). Next, we constructed PPI network, and cytoHubba was employed to select the top 10 hub nodes, including PLK1, DLGAP5, KIF23, BIRC5, AURKB, CDCA8, RRM2, BUB1B, UBE2C, and CDC20 (Figure 6). KIF23, a member of kinesin proteins, and its role in tumor development has been demonstrated in many tumors (Gao et al., 2020; Hu et al., 2020), yet its role in ccRCC remains elusive. Therefore, we explored the expression and function of KIF23 in ccRCC.

### Kinesin Family Member 23 was Significantly Elevated in Clear Cell Renal Cell Carcinoma

**FIGURE 7:**
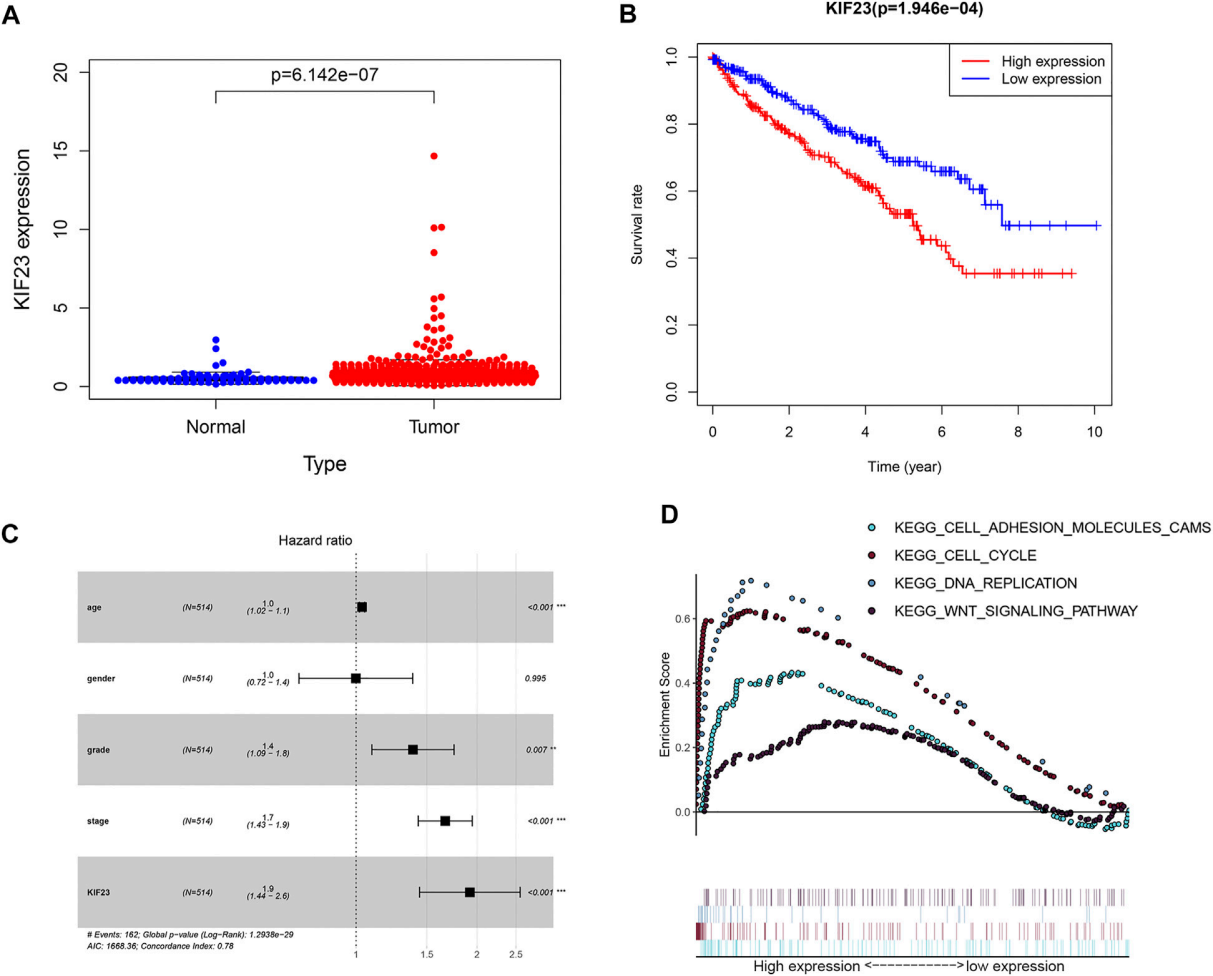
The expression data of KIF23 in ccRCC from TCGA database. **(A)** Human KIF23 expression levels in normal and ccRCC tissues from the TCGA database. **(B)** Kaplan-Meier plotters of KIF23 in ccRCC. **(C)** Multivariate Cox hazards regression analyses. **(D)** GSEA analysis.

**FIGURE 8:**
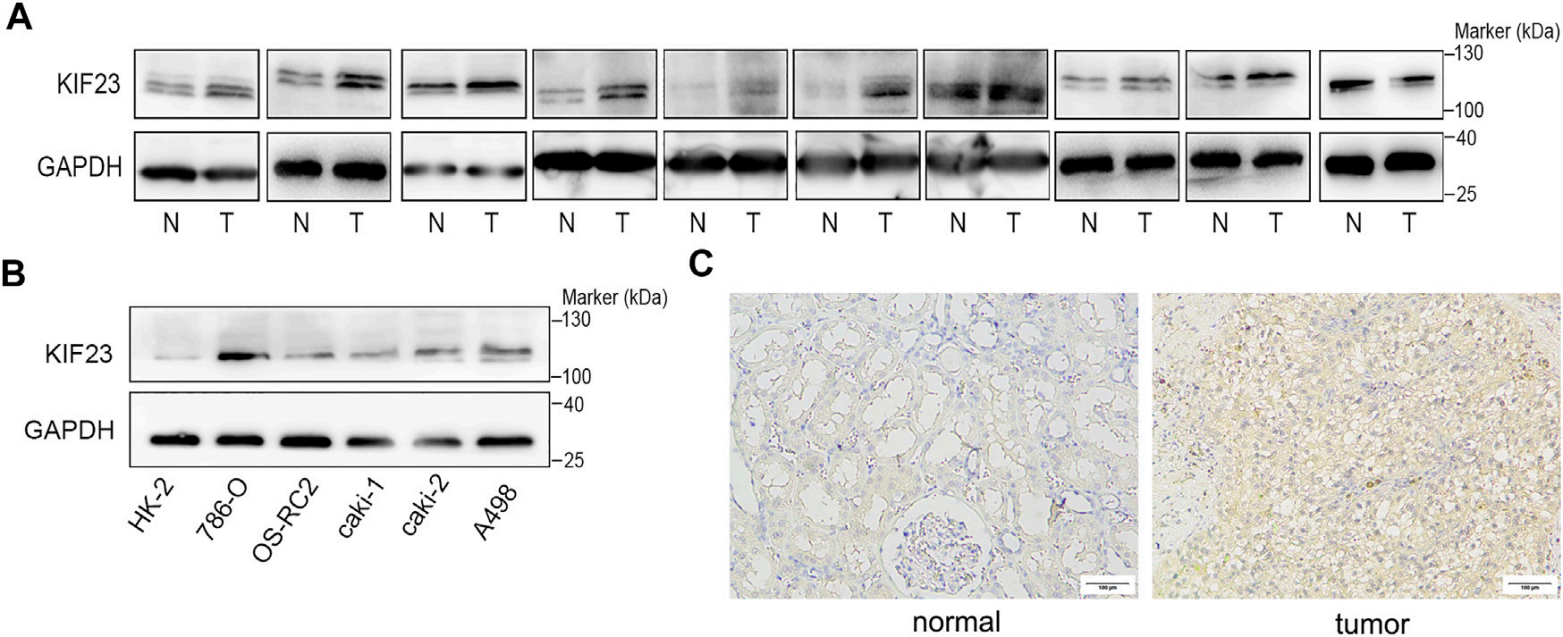
KIF23 is overexpressed in ccRCC cell lines and tissues. **(A)** The expression level of KIF23 in 10 paired ccRCC samples. **(B)** KIF23 protein levels in ccRCC cell lines (786-O, OS-RC-2, caki1, caki2, and A498) and HK-2 were assessed using Western blot assays. **(C)** Immunohistochemistry staining of KIF23 expression level in ccRCC samples.

The TCGA database showed that KIF23 mRNA was significantly elevated in ccRCC tissues (Figure 7A). And patients with high expression of KIF23 showed worse OS rates (Figure 7B). Univariate analysis showed no significant difference in survival based on gender. KIF23 expression level, age, tumor stage and grade were associated with OS in ccRCC patients (Supplementary Table S1). Multivariate Cox risk regression analysis showed that KIF23 was an independent factor of OS (HR = 1.919, 95% confidence interval, 1.439–2.560, *p* < 0.001) (Figure 7C, Supplementary Table S2). Moreover, results from GSEA analysis showed that cell adhesion molecules (CAMs), DNA replication, cell cycle and Wnt signaling pathway were significantly enriched in the KIF23 high-expression group (Figure 7D). As anticipated, the protein level of KIF23 was also increased in ccRCC tumor tissues (Figure 8A). The protein expression level of KIF23 was detected in HK-2 cells and five human ccRCC cell lines. As showed in Figure 8B, KIF23 was increased in ccRCC cell lines compared with HK-2 cells. Moreover, the results of immunohistochemistry staining showed that KIF23 expression were higher in tumor specimens (Figure 8C).

### Kinesin Family Member 23 Promotes the Malignant Behavior of Clear Cell Renal Cell Carcinoma Cells

**FIGURE 9:**
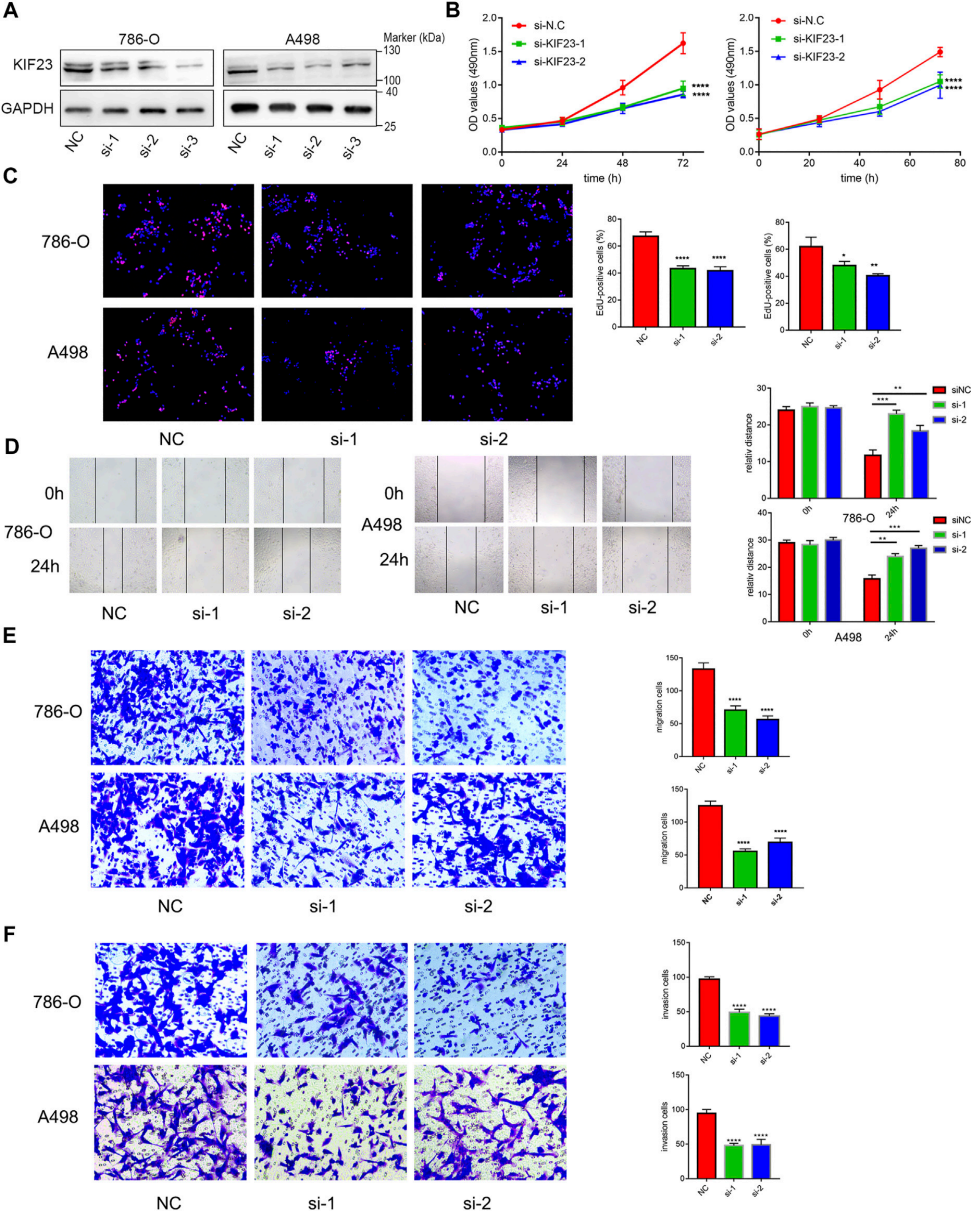
KIF23 promotes proliferation, migration and invasion of ccRCC cells. **(A)** Protein expression levels of KIF23 in 786-O and A498 cells following siRNA interference. **(B,C)** CCK-8, EdU assays. **(D–F)** Wound-healing, transwell migration and invasion assays.

**FIGURE 10:**
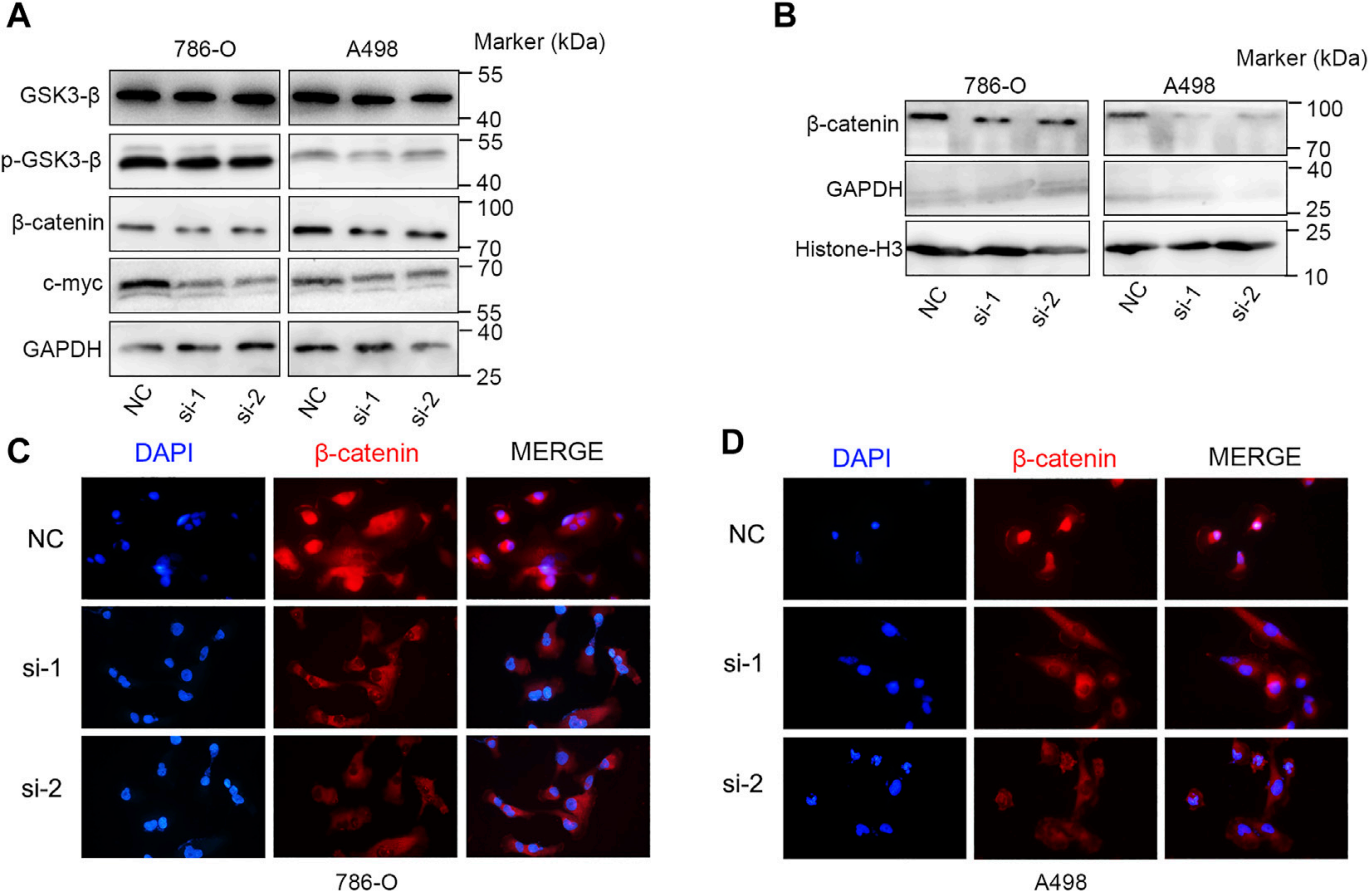
KIF23 knockdown inhibits the Wnt/β-catenin signaling pathway. **(A)** KIF23 knockdown reduced the levels of p-GSK3-β, β-catenin and c-Myc. **(B)** KIF23 knockdown reduced β-catenin levels in the nucleus. **(C,D)** Immunofluorescence staining of β-catenin in 786-O and A498 cells.

We further explored the role of KIF23 on the proliferation, migration and invasion of ccRCC cells. Silencing of KIF23 expression using siRNA resulted in down-regulation of KIF23 protein in 786-O and A498 (Figure 9A). Compared with the negative control (NC) group, knockdown of KIF23 expression inhibited the proliferation of ccRCC cells as measured by CCK-8 and EdU assays (Figures 9B,C). The migration and invasion ability were further measured using wound-healing assay and transwell assays. The results showed that interfering with KIF23 expression reduced the migration and invasion ability of ccRCC cells (Figures 9D,E). To further elucidate the mechanisms underlying KIF23-promoted ccRCC proliferation and migration, GSEA analysis was used to predict the downstream pathways, and Wnt signaling pathways were enriched in the KIF23 high-expression group (Figure 7D). We found that KIF23 knockdown decreased the expression levels of molecules on the Wnt signaling pathway include p-GSK3-β, β-catenin and c-Myc (Figure 10A). Moreover, analysis of subcellular protein fraction showed that knockdown of KIF23 reduced nuclear translocation of β-catenin (Figure 10B). Immunofluorescence staining was used to assess β-catenin translocation, and it was found that knockdown of KIF23 expression reduced nuclear localization of β-catenin in 786-O and A498 cells (Figures 10C,D).

## Discussion

Advanced RCC has a poor prognosis due to its resistance to conventional radiation therapy and chemotherapy (Santoni et al., 2013). With the increasing understanding of RCC biology, many therapies have been introduced. Tyrosine kinase monoclonal antibodies or inhibitors targeting vascular endothelial growth factor (VEGF) have showed a better therapeutic effect on RCC (Motzer and Molina, 2009). mTOR inhibitors also have a remarkable survival advantage (Hudes et al., 2007). Despite the use of novel targeted therapeutic agents, metastatic RCC will eventually progress owing to primary or secondary drug resistance (Iacovelli et al., 2014). Metastasis is the most important reason affecting patient prognosis in ccRCC patients, and analyzing transcriptome differences of metastatic tumors will help to identify factors affecting metastasis and predict clinical outcomes. The present study sought to address these gaps in knowledge by comparing transcriptome profiles between metastatic and primary tumors, and 74 genes were identified as metastasis-associated genes. Furthermore, 54 genes associated with the prognosis of ccRCC patients were screened.

In a prognostic model of metastasis-related genes based on LASSO Cox regression analysis, we found a shorter survival time in the high-risk score group. Since the tumor immune microenvironment has a non-negligible impact on tumor progression, we analyzed differences in the immune components of the high- and low-risk groups. ccRCC in the low-risk group was associated with higher proportions of resting memory CD4 T cells, activated NK cells, monocytes, resting DCs, and resting mast cells. This is similar to previous studies showing that accumulation of CD8 T lymphocytes and NK cells in tumors implies favorable clinical outcomes (Fridman et al., 2012). DCs are antigen-presenting cells that play an important role in promoting antitumor immune responses. Current studies suggest that DCs infiltration is an important prognostic parameter, which usually depends on cellular localization and cellular maturation (Eisenthal et al., 2001; Sandel et al., 2005). Overall, our results indicate that there are different immunobiological processes and pathways between the two groups. Immune heterogeneity between two groups may be an important factor driving the differences observed in OS.

Gene expression profiles also differed between the two groups, which may affect ccRCC progression, response to treatment and prognosis. DEGs were associated with different biological processes. GO enrichment analysis indicated that DEGs were enriched in “extracellular matrix organization”, “extracellular structure organization” and “positive regulation of secretion by cell” in the “biological process” category, Previous studies found that extracellular matrix (ECM) is a complex network providing structural and guiding clues to surrounding cells (Naba et al., 2017). The ECM triggers tumor progression by affecting cell proliferation and metastasis. In addition, abnormal ECM also affects stromal cell behavior, such as inflammation and angiogenesis, which promote the formation of tumorigenic microenvironment (Cukierman and Bassi, 2010; Pickup et al., 2014). ECM protein is also considered to be an important part of the metastatic niche, which can maintain the characteristics of cancer stem cells and promote the growth of metastatic cells (Malanchi et al., 2011; Oskarsson et al., 2011). Tumor ECM induced tumor cell metastasis through extracellular proteases such as matrix metalloproteinases (MMPs) (Wang et al., 2021). PLK1, DLGAP5, KIF23, BIRC5, AURKB, CDCA8, RRM2, BUB1B, UBE2C, and CDC20 were identified as the hub genes. Multiple members of the KIF family have been shown to promote ccRCC metastasis, invasion and drug resistance (Li et al., 2019a; Jin et al., 2019; Ren et al., 2020). As a member of KIF family, KIF23 promotes ovarian tumor proliferation, cell cycle progression and is closely related to metastasis. In glioma, silencing KIF23 inhibits the proliferation of glioma cells (Takahashi et al., 2012; Li et al., 2019b). In ccRCC, we found the expression of KIF23 was significantly elevated in tumor tissues. Its expression level correlates with patient prognosis. In ccRCC tumor biology, knocking down KIF23 expression in ccRCC cells inhibited the malignant behavior of ccRCC cells. Therefore, KIF23 may be an important molecular marker promoting the progression of ccRCC.

Abnormal Wnt signaling is involved in many human tumors (Nwabo Kamdje et al., 2014; Cojocaru et al., 2015; Xu et al., 2016). β-catenin is an executor of the Wnt signaling. β-catenin is phosphorylated by a “degradation complex” in the absence of Wnt ligands, phosphorylated β-catenin will be further degraded by ubiquitination to inhibit the activation of Wnt signaling (Bhanot et al., 1996). When the Wnt signaling is activated, the binding of Wnt protein phosphorylate and inactivate GSK3-β in cells, inhibit the formation of degradation complexes and cause accumulation of β-catenin and promote its nucleus translocation (Behrens et al., 1998). β-catenin forms the β-catenin-TCF4 complex in the nucleus and activates the transcription of multiple oncogenes including c-Myc and cyclin D1 (Morin et al., 1997; Vallée et al., 2017). Our study found that knockdown of KIF23 expression in ccRCC inhibited the phosphorylation of GSK3-β, decreased the expression level of β-catenin and its nuclear translocation and thus inhibited the expression of c-Myc, thereby regulating the malignant behavior of ccRCC cells.

In conclusion, based on the construction of prognostic signatures of metastasis-related genes, we identified KIF23 as an important gene that drives ccRCC progression and affects patient prognosis. By knocking down KIF23 expression, we found that KIF23 enhanced the proliferation, migration and invasion capacity of ccRCC cells by regulating the nuclear translocation of β-catenin. Inhibition of KIF23 expression or function is a potential therapeutic approach for ccRCC.

## Data Availability

Publicly available datasets were analyzed in this study. This data can be found here: The microarray datasets GSE85258 and GSE105288 were obtained from the National Center for Biotechnology Information (NCBI) Gene Expression Omnibus (GEO) database. The gene expression profiles of 530 ccRCC were downloaded from the TCGA data portal (https://tcga-data.nci.nih.gov/tcga/).
