# Supplementary material for: Identification of KIF23 as a Prognostic Biomarker Associated With Progression of Clear Cell Renal Cell Carcinoma
Source: Front Cell Dev Biol. 2022 Apr 11;10:839821. doi: 10.3389/fcell.2022.839821 (PMC9035542; doi:10.3389/fcell.2022.839821)
Supplement: Supplementary file 1 [file Table1.DOCX]

| id | HR | HR.95L | HR.95H | pvalue |
| --- | --- | --- | --- | --- |
| age | 1.029566 | 1.015941 | 1.043375 | 1.81E-05 |
| gender | 0.961378 | 0.697784 | 1.324547 | 0.809633 |
| grade | 2.327644 | 1.882296 | 2.878362 | 6.32E-15 |
| stage | 1.940875 | 1.694659 | 2.222862 | 9.61E-22 |
| KIF23 | 2.401521 | 1.901807 | 3.032539 | 1.84E-13 |
