# Supplementary material for: Identification of KIF23 as a Prognostic Biomarker Associated With Progression of Clear Cell Renal Cell Carcinoma
Source: Front Cell Dev Biol. 2022 Apr 11;10:839821. doi: 10.3389/fcell.2022.839821 (PMC9035542; doi:10.3389/fcell.2022.839821)
Supplement: Supplementary file 2 [file Table2.DOCX]

| id | HR | HR.95L | HR.95H | pvalue |
| --- | --- | --- | --- | --- |
| age | 1.034915 | 1.01956 | 1.050502 | 6.80E-06 |
| gender | 0.998861 | 0.7211 | 1.383613 | 0.99453 |
| grade | 1.385382 | 1.094828 | 1.753046 | 0.00664 |
| stage | 1.666987 | 1.42812 | 1.945808 | 9.42E-11 |
| KIF23 | 1.919314 | 1.43878 | 2.560341 | 9.24E-06 |
